# Energy-Efficient Control with Harvesting Predictions for Solar-Powered Wireless Sensor Networks

**DOI:** 10.3390/s16010053

**Published:** 2016-01-04

**Authors:** Tengyue Zou, Shouying Lin, Qijie Feng, Yanlian Chen

**Affiliations:** College of Mechanical and Electronic Engineering, Fujian Agriculture and Forestry University, Fuzhou 350002, China; linshouying@fafu.edu.cn (S.L.); qijiefeng@139.com (Q.F.); petereisenhower@hotmail.com (Y.C.)

**Keywords:** wireless sensor network, solar cells, energy prediction, shadow detection

## Abstract

Wireless sensor networks equipped with rechargeable batteries are useful for outdoor environmental monitoring. However, the severe energy constraints of the sensor nodes present major challenges for long-term applications. To achieve sustainability, solar cells can be used to acquire energy from the environment. Unfortunately, the energy supplied by the harvesting system is generally intermittent and considerably influenced by the weather. To improve the energy efficiency and extend the lifetime of the networks, we propose algorithms for harvested energy prediction using environmental shadow detection. Thus, the sensor nodes can adjust their scheduling plans accordingly to best suit their energy production and residual battery levels. Furthermore, we introduce clustering and routing selection methods to optimize the data transmission, and a Bayesian network is used for warning notifications of bottlenecks along the path. The entire system is implemented on a real-time Texas Instruments CC2530 embedded platform, and the experimental results indicate that these mechanisms sustain the networks’ activities in an uninterrupted and efficient manner.

## 1. Introduction

Wireless sensor networks (WSNs) consist of several embedded devices, known as sensor nodes, which are used to measure environmental phenomena in real time and send data back to workstations through a wireless component. Without a wire connection, they are suitable for use in applications under harsh environment conditions, such as inpatient care [1], smart buildings [2], coal mining [3], bridge monitoring [4] and agricultural production [5]. A sensor node is typically powered by a limited-capacity lithium battery, which supplies the circuitry by providing the current required to sustain each component of the node. The total energy consumption is the sum of each component on a node (e.g., sensor, microcontroller and radio), and each part may operate at different states of energy. Hence, the lifetime of a sensor node is the time taken to discharge its battery below a sustainable level for operation.

Numerous studies have been conducted to develop energy harvesting systems for powering the sensor nodes for a potentially infinite node lifetime [6], and solar cells are a typical solution [7]. Unfortunately, in real-world environments, the energy supplied by such a harvesting system is generally intermittent and insufficient for all components of the node. Thus, batteries are still indispensable for compensating power when the energy production of the harvesting system is insufficient. However, the battery level may be too low to maintain node function, and the harvesting system may be unable to provide sufficient energy to recharge the battery, which could lead to the temporary unavailability of the node and cause the entire WSN to be non-operational.

**Figure 1:**
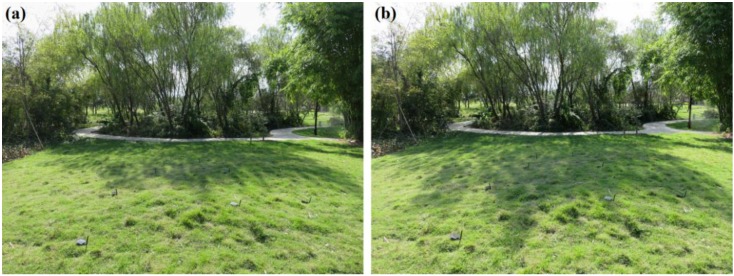
(**a**) Illustration of a shadow region at some time; (**b**) Illustration of the moving and shape-changing shadow region half an hour later.

In this report, we consider a WSN for environmental monitoring, in which the sensor nodes are exposed to sunshine to harvest energy. However, under actual conditions, the node may be sheltered from the sun by the shadow of a building or a tree. The shadow region is not static, and it moves and changes its shape based on the incidence angle of the sun. Figure 1 depicts an illustration of the moving and changing shadow situation for the WSN. In Figure 1a, there are only four sensor nodes covered by the shadow cast by the trees, whereas after half an hour, seven nodes are covered by the shadow region. The area and the shape of the shadow increased over 30 min, which may lead to a sudden significant decrease in the harvested energy and affect the scheduling plan of the node system. Thus, shadow detection and movement prediction methods are required to avoid this risk.

To control the energy consumption and achieve a longer lifetime, we built a model to describe the energy status of the sensor node and used suitable methods for harvesting prediction, shadow detection, task scheduling, and routing optimization. The primary contributions of this report can be described as follows: (1) a piecewise least squares curve fitting with extended Kalman filter is introduced for harvested energy prediction on a sensor node to improve its task schedule; (2) shadow detection and movement prediction algorithms are proposed to avoid an energy risk; (3) node clustering and routing algorithms with bottleneck warnings at structural level are introduced to optimize the data transmission; and (4) methods are implemented on a Texas Instruments (TI, Dallas, TX, USA) CC2530 WSN platform to validate their effect. The remainder of this report is organized as follows: after reviewing related studies in Section 2, we provide a node energy management model in Section 3. Section 4 describes the task schedule method based on energy prediction using shadow detection. In Section 5, we present clustering and routing methods for saving energy at the nodes. Experiments are performed on real-time embedded hardware implemented on the TI CC2530 platform, and the results are presented in Section 6. Finally, the conclusions are stated in Section 7.

## 2. Related Work

Green computing is a new term that refers to minimizing the negative impact of equipment on the environment. The concept of self-powered wireless sensor networks using green energy has attracted a lot of attention in recent years [8], especially in opportunistic routing [9] and energy control [10]. Solar power is an important type of green energy that can be acquired from sunshine to support WSNs in the wild. However, because the energy harvested from the sun is unstable and depends considerably on the weather, dynamic power management [11,12] should be considered to solve this problem. Due to the fact that power modelling is the first issue in energy management, SIVEH [13] and EFCon [14] were introduced to describe the energy harvesting and consuming conditions of the sensor nodes. Furthermore, the task schedules could be adjusted based on the real-time energy levels. The Prediction FREE Energy Neutral (P-FREEN) method [15], which is based on budget assigning principles, and the adaptive packet transmission period method [16] were proposed to improve the energy efficiency. Additionally, a few studies attempted to extend the network lifetime using advanced hardware design, e.g., PWM control [17] and Maximum power Point Tracking (MPPT) [18]. Most of these mechanisms only consider the remaining battery level or harvested energy at the right time for the task plan; however, the energy acquired in the future will also influence the trends of the total energy. Some studies have shown that the Exponentially Weighted Moving Average (WCMA) is a low computational algorithm with relatively high accuracy using simulation [19]. In this study, we predict the energy on sensor node hardware with a piecewise least squares curve fitting, which may result in energy use being more targeted and anticipatory.

Data transmission is another primary task in WSNs, and it is more energy-consuming than sampling tasks. A data transmission reduction strategy is one of the few ways to reduce energy consumption [20,21]. Hence, reducing the consumption related to clustering and routing plays an important role in several research plans. Low-Energy Adaptive Clustering Hierarchy (LEACH) [22] is a clustering protocol that utilizes randomized rotation of clusterheads to distribute the energy load. However, it only performs well under the homogeneous network, but poorly in heterogeneous environments. The Hybrid Energy-Efficient Distributed clustering (HEED) [23] is another distributed clustering algorithm which selects the clusterheads according to the residual energy of each node. But under heterogeneous environments, the low-energy nodes may own larger election probability than the high-energy ones in HEED. The Stable Election Protocol (SEP) scheme [24] is designed for two-level heterogeneous wireless sensor networks, which is composed of two types of nodes according to different initial energy. The advanced nodes have more energy than the normal ones at the beginning. The SEP is not fit for widely used multi-level heterogeneous wireless sensor networks due to the fact that it includes two types of nodes [25]. The Deterministic Energy-efficient Clustering Protocol (DEC) [26] and Distributed Energy-Efficient Clustering algorithm (DEEC) [25] are two self-organizing clustering protocols for heterogeneous networks. The DEC uses the sensor node’s residual energy solely as the election criterion, while the DEEC takes the ratio between residual energy of each node and the average energy of the network into consideration. These two methods both use the residual energy without the consideration of the variation trend of harvested energy in an energy harvesting system. Enhanced developed Distributed Energy-Efficient Clustering (EDDEEC) [27] was introduced to prolong network lifetime. Unlike previous methods, this method considers the effects of the radio environment and changes the clusterhead selection probability in a dynamic manner. The simulation results confirm the performance of the method. The Distributed Clustering Protocol Using Voting and Priority (DCPVP) [28] method, which is based on the mean distance from the neighbours and the remaining energy, can decrease the construction time as well as the energy consumption of the clustering process in sensor networks. The simulation results confirm its effectiveness with limited resources and battery-powered nodes in harsh and inaccessible environments. Unfortunately, there are no experimental data to further validate that the computational complexity of these methods can be handled by sensor nodes with limited computing ability.

Advanced routing algorithms have also been developed for energy savings. Wastage-aware routing [29] is a route selection scheme that considers energy wastage in addition to residual battery power and forecasts the energy harvest information of the nodes. Zone-based routing [30] is another novel method that uses a parallel and distributed broadcasting technique to reduce redundant transmission and save energy. Meanwhile, the intelligent energy protocol [31] uses reinforcement-learning techniques to enhance its effects. Recently, the ant colony algorithm was used to optimize the deployment of sensor nodes in the network [32]. Furthermore, an energy-efficient topology control mechanism [33] could be an important contribution to transmission power savings. An appropriate task scheduling plan could also markedly prolong the lifetime of the network, and weather forecasting [34] and Quality of Service (QoS) [35] could be involved in this optimization. All of these methods are beneficial for improving energy efficiency but may not be deployed successfully on sensor nodes due to limited energy and computing abilities. Thus, in this study, we use a series of simple algorithms for efficient clustering and routing on real-time hardware. The experimental results indicate their contribution to energy savings.

## 3. Energy Harvest and Consumption Models

We consider a WSN for environmental monitoring applications, especially in agriculture and wooded areas. The sensor nodes are powered by lithium batteries, which are recharged using solar cells. The current is drained from the batteries based on the applications under which the nodes are operating [12]. To analyse the harvesting situation and battery consumption, relative models need to be constructed for the solar cells and lithium batteries on a sensor node.

### 3.1. Solar Cells

Solar cells are composed of semiconductor materials in a crystalline state and can convert the energy of photons into electricity via the photovoltaic effect. The efficiency of a solar cell (σ) is defined as a measure of the conversion efficiency for the incident light absorbed by a solar cell converted into electric power, which can be calculated as follows:

(1) $$\sigma =S_{\max}/D\times S$$

where *S_max_* is the maximum output power provided by the solar cell (in Watts); *D* is the irradiance, defined as the density of the incident power on a surface (in W/m^2^); and S is the surface area of the solar cell (in m^2^). In our study, we selected the KINGRO-004V solar cell (KINGRO, Shaoxing, China), which is a 70 × 70 mm^2^ monocrystalline cell that provides a maximum current of 120 mA. Table 1 lists the specifications of this cell.

**Table 1:** Specifications of the KINGRO-004V solar cell.

Parameter	Value
Specified load voltage (*V_ld_*)	4 V
Typical current at V_ld_ (*I_ld_*)	120 mA
Open circuit voltage (*V_oc_*)	4.5 V
Short circuit current (*I_oc_*)	130 mA
Maximum output power (P_max_)	500 mW
Surface area (*S*)	49 cm^2^

The solar cell efficiency depends on the brightness conditions of its location, its geographical coordinates, the solar time, and its inclination with respect to the Sun’s rays. In this study, we attempted to keep the cell positioned approximately perpendicular to the Sun’s rays to maximize efficiency.

### 3.2. Lithium Battery

**Figure 2:**
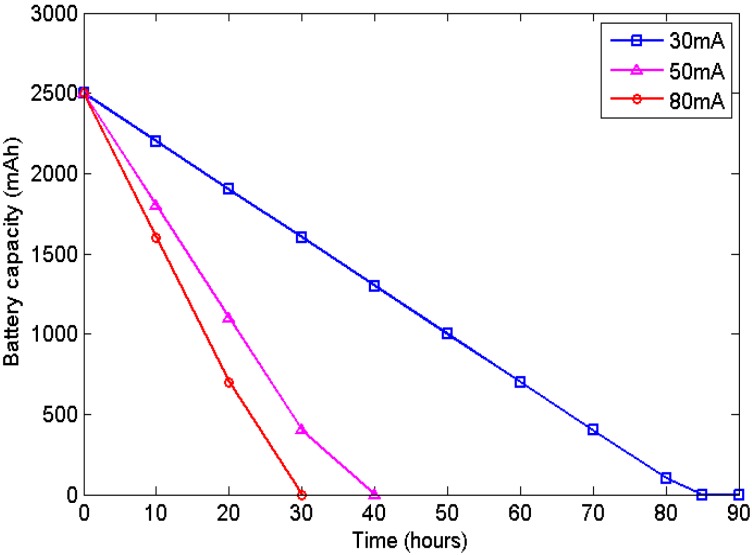
Illustration of battery discharge.

The battery discharge condition depends primarily on the power requirements of the sensor node. In this study, we use a LP903158 lithium battery manufactured by ZONCELL Corporation (Shenzhen, China) with a storage capacity of 2500 mAh on our sensor node. Furthermore, Table 2 presents the specifications of this battery. To illustrate the battery discharge procedure, we select three typical sample tasks for the sensor node. The battery consumption procedures are illustrated in Figure 2, with average current draws of 30, 50 and 80 mA, respectively. Furthermore, in this study, the batteries are charged from the solar cell when the harvested energy is sufficient.

**Table 2:** Specifications of the LP903158 lithium battery.

Parameter	Value	Parameter	Value
Battery capacity	2500 mAh	Maximum discharge current	1 C
Nominal voltage	3.7 V	Discharge cutoff voltage	2.75 V
Product weight	30 g	Discharge temperature	−10 °C~+60 °C
Standard charge current	0.2 C	Charge and discharge times	≥ 800
Maximum charge current	0.5 C	Self-discharge current	≤ 400 µA
Continuous discharge current	0.5 C		

### 3.3. Energy Model

Let *P_max_* represent the maximum battery capacity of the WSN node and *P_min_* be the minimum capacity necessary to sustain the node operation. Without considering the power leakage, the energy production and consumption of a sensor node can be described using Equation (2) as follows [12]:

(2) $$P_{t}=P_{0}+\theta \int_{0}^{t}{\left[E_{h}(t)-E_{c}(t)\right]}^{+}-\int_{0}^{t}{\left[E_{c}(t)-E_{h}(t)\right]}^{+}dt\forall t\in [0,\infty )$$

where *P_t_* denotes the battery power level at time *t*, *E_h_(t)* represents the energy produced by the solar harvesting component at time *t*, *E_c_(t)* is the energy consumed by the sensor node at time *t*, and θ∈[0,1) denotes the charging efficiency of the battery. The rectifier function ${\left[x\right]}^{+}$in the formula can be defined using Equation (3) as follows:

(3) $${\left[x\right]}^{+}=\{\begin{array}{c} x,x\geq 0\\ 0,x<0 \end{array}$$

The first part of this formula denotes the initial energy of the battery, and the second term accounts for the energy needed to recharge the battery, which is produced by the solar cell and not consumed. Moreover, the last term represents the power drained from the battery by the sensor node. Additionally, the energy model considers that the battery is not an ideal energy tank and that it may dissipate part of the redundant energy as heat. Once this energy model is constructed, energy management and prediction for sensor nodes can be conducted.

## 4. Harvest Prediction and Energy Control

**Figure 3:**
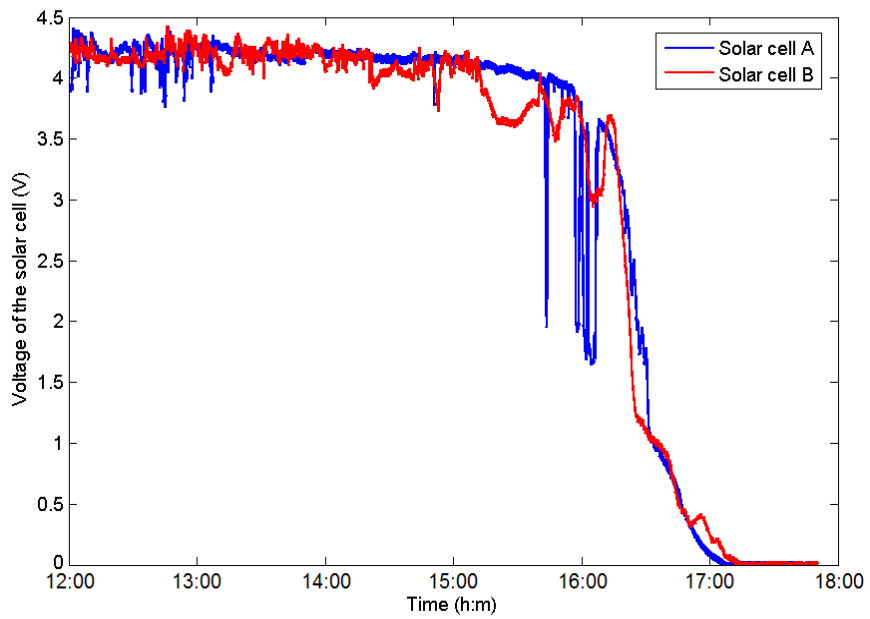
Illustration of the harvested energy of two solar cells at different locations.

Due to its high energy density, solar energy is a popular kind of energy harvested from Nature, being widely utilized. However, the energy produced by the solar cell depends on the weather, location and time of day, and it is typically difficult to precisely predict [14]. Figure 3 indicates the energy curve harvested by two solar cells located at different places from noon to night. It is evident that the solar cell cannot supply stable energy for equipment because it is considerably influenced by the sunlight intensity and location. The voltage of the cells increases gradually in the morning, peaks at noon due to direct sunlight conditions, and then declines in the afternoon; it may fluctuate due to the passage of clouds across the sky and shadows across the ground. Furthermore, the voltage does not depend linearly on the luminous intensity. When the solar cell reaches saturation conditions, the energy cannot increase, even if the solar radiation is yet to reach its peak value (which occurs at 12:00).

Additionally, location plays an important role in a solar-powered system. Solar cells deployed at different places will be influenced by the environment at their positions and then produce different harvesting energies. The shadows of trees or buildings constitute a primary reason for the difference in energies. The shadow shields the solar cell from sunshine and decreases the voltage. Thus, when a node is shielded by a shadow, it should be slowed to save energy. However, the position of a shadow is not static, and its position and area vary according to the angle of incidence of the sunlight. Therefore, it is difficult to estimate which node should be set to save energy and which should be removed from the energy saving group at the next interval. In this section, we propose a novel estimation algorithm to track the shadow based on the correlation between the region and the harvesting energy.

### 4.1. Time Correlation Prediction

Because the energy harvested from the solar cell is greatly influenced by the luminous intensity, it is possible to develop an energy-consumption plan by predicting the location of the sunlight at the next time point. By reducing the current consumption before sunset or bad weather, the WSN node can remain active for a longer duration and operate more efficiently. Because the volume of solar radiation exhibits continuity over time, a piecewise least squares curve fitting estimation [36,37] based on previous sensing data is used to predict sunlight conditions in the future. Because the real-time voltage level of the solar cell is positively correlated with the harvested current energy, it can be viewed as an observation of the solar energy. There are several base formulas for fitting, such as linear functions, quadratic functions, cubic functions, fourth-order polynomial functions, fifth-order polynomial functions, B-spline curves and Bezier curves. However, the computational ability of the sensor node is limited; thus, the selected formula should be efficient and save time.

**Figure 4:**
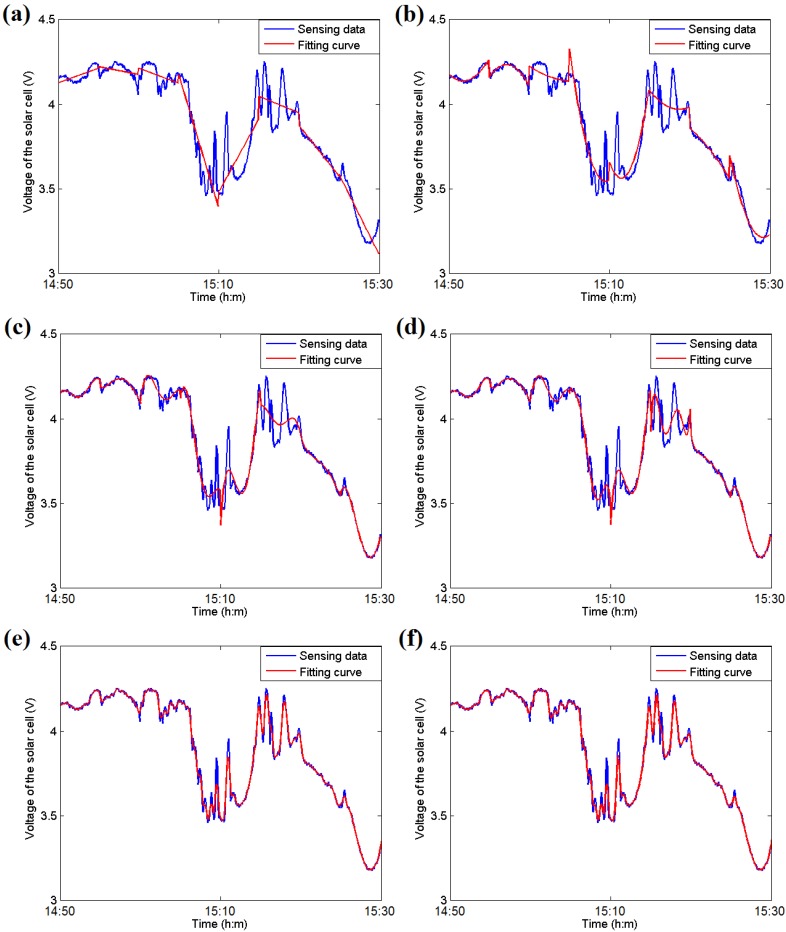
(**a**) An example of the piecewise linear curve fitting estimation; (**b**) An example of the piecewise quadratic curve fitting estimation; (**c**) An example of the fourth-order polynomial curve fitting estimation; (**d**) An example of the fifth-order polynomial curve fitting estimation; (**e**) An example of the B-spline curve fitting estimation; (**f**) An example of the Bezier curve fitting estimation.

Figure 4 illustrates the experimental results of the fitting estimation for six different formulas. The sensor data are acquired from the solar cell on a node in an open lawn from 14:10 to 15:30 in October in Fuzhou, China. The evaluation is performed on a computer with an Intel i5 3.4 GHz CPU and 4 GB memory using MATLAB. Piecewise fitting for each 5-min section is applied to the curve after using a mean filter with a window parameter of 25 s.

**Figure 5:**
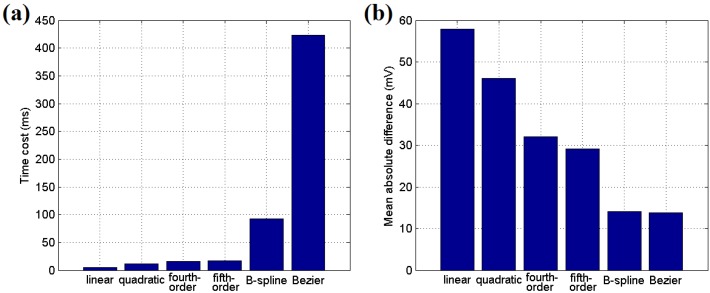
(**a**) Time cost of the fitting methods; (**b**) Mean absolute difference for the fitting methods.

As illustrated in Figure 4, the fourth-order or fifth-order polynomial functions provide better approximations than the linear and quadratic functions, and their mean absolute differences (MADs) are lower than those of the linear and quadratic methods. Moreover, the B-spline and Bezier curves have the lowest MAD values in the fitting procedure, as indicated in Figure 5b, but they require considerably more time than the polynomial methods. Due to the limited computational and storage abilities of a sensor node, the fifth-order polynomial functions, which have a relatively low time cost and MAD value, are most suitable for use at an embedded node.

### 4.2. Extended Kalman Filter (EKF)

As illustrated in Figure 3, the energy acquired from the environment varies strongly with the luminous intensity, and the voltage of the solar cell can be viewed as an observation. However, due to noise, the observation cannot reflect the actual energy conditions at the right time. To improve the robustness of the sampling data, filter tools should be used to smooth the curve before prediction. A mean filter, also known as a linear filter, is the most commonly used filter tool, and the basic principle is to replace the original value of each point with the mean value within a short scope. In the previous section, it was used to smooth the curve before the fitting estimation. However, the mean filter only reduces the fluctuation within a certain range and cannot decrease the process and observation noise. Thus, a more powerful Kalman filter is considered to solve this problem.

The Kalman filter [38] is a recursive algorithm that uses a series of noisy measurements over time to yield optimal estimates of the states of a linear stochastic process. It can minimize the mean square of the estimation error under white noise. Because of its low computational load and optimal performance, it is widely used in digital systems and online applications. The standard Kalman filter involves a two-step process that consists of predicting and updating. In the predicting step, the filter produces estimates of the current state variables ${\hat{x}}_{k}^{-}$ along with an *a priori* estimate of their covariance ${P_{k}}^{-}$. The updating step refreshes the a posteriori estimate state $\hat{x}$ and the a posteriori estimate covariance $P_{k}$ based on the *a priori* estimates. Because the algorithm only uses the current input measurements, the previously calculated state and its uncertainty matrix, no additional past information is required; thus, it can be used in real-time applications.

**Figure 6:**
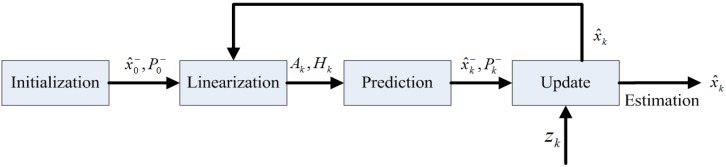
Estimation procedure of the extended Kalman filter (EKF).

The standard Kalman formulas can only address linear problems; however, the voltage tested from the solar cell does not follow a linear relationship over time. Thus, an extended Kalman filter (EKF) [39,40] is selected to adjust this nonlinear curve. As depicted in Figure 6, the EKF first constructs a linear system to approximate the nonlinear system near the current estimated state, and the standard Kalman filter equations can then be used for the linearized state. Equations (4) and (5) describe the nonlinear process in the form of discrete stochastic differential equations as follows:

(4) $$x_{k}=f(x_{k-1},u_{k-1},w_{k-1})$$

(5) $$z_{k}=h(x_{k},v_{k})$$

where *x_k_* represents the process state at time *k*; w_k_ and v_k_ denote the excitation noise and the observation noise*,* respectively; *z_k_* is the observed variable; and *u_k_* is the control vector at time *k*.

The entire EKF estimation procedure consists of four steps: initialization, linearization, prediction and update. In the first step, the initial values are assigned to ${\hat{x}}_{k}^{-}$ and $P_{k}^{-}$ for further estimation, where the symbol—represents the *a priori* element and the symbol ^ denotes the estimation value.

After initialization, the linearization step is used to develop an approximate linear process for the nonlinear system. The prediction step can then be used to estimate the *a priori* value of x using Equations (6) and (7) as follows:

(6) $${\hat{x}}_{k}^{-}=f({\hat{x}}_{k-1},u_{k-1},0)$$

(7) $$P_{k}^{-}=A_{k}P_{k-1}A_{k}^{T}+W_{k}Q_{k-1}W_{k}^{T}$$

where the notation ${\hat{x}}_{k}^{-}$ represents the estimate of x at time k given observations up to and including time k − 1; $A_{k}$ is the state transition model, which is applied to the previous state ${\hat{x}}_{k}^{-}$; $P_{k}^{-}$ denotes the *a priori* estimate covariance at time k given observations up to and including time k − 1; Q_k_ represents the covariance of the process noise, which is assumed to be drawn from a zero mean multivariate normal distribution; W_k_ is the Jacobian matrix of the process at time k; and u_k_ is the control vector at time k:

(8) $$P_{k}=(I-K_{k}H_{k})P_{k}^{-}$$

(9) $$K_{k}=P_{k}^{-}H_{k}^{T}{\left(H_{k}P_{k}^{-}H_{k}^{T}+V_{k}R_{k}V_{k}^{T}\right)}^{-1}$$

(10) $${\hat{x}}_{k}={\hat{x}}_{k}^{-}+K_{k}(z_{k}-h({\hat{x}}_{k}^{-},0))$$

Here, ${\hat{x}}_{k}$ is the a posteriori state estimate; $P_{k}$ is the a posteriori estimate covariance at time k given observations up to and including time k; z_k_ is the observation of the true state x_k_; K_k_ is the Kalman gain at time k; H_k_ and V_k_ are the Jacobian matrices of the observations, which map the true state space onto the observed space; and R_k_ denotes the covariance of the observation noise, which is assumed to be zero mean Gaussian white noise.

**Figure 7:**
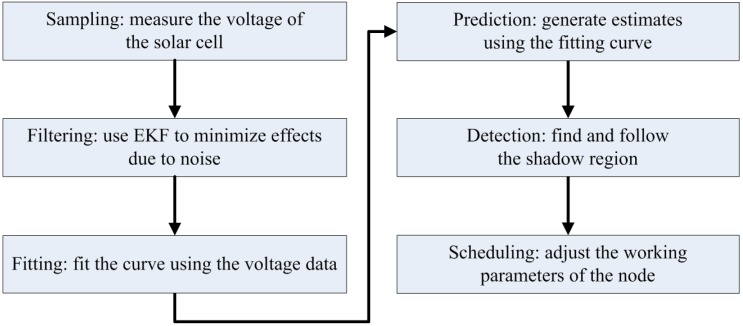
Prediction procedure of the solar harvested energy.

**Figure 8:**
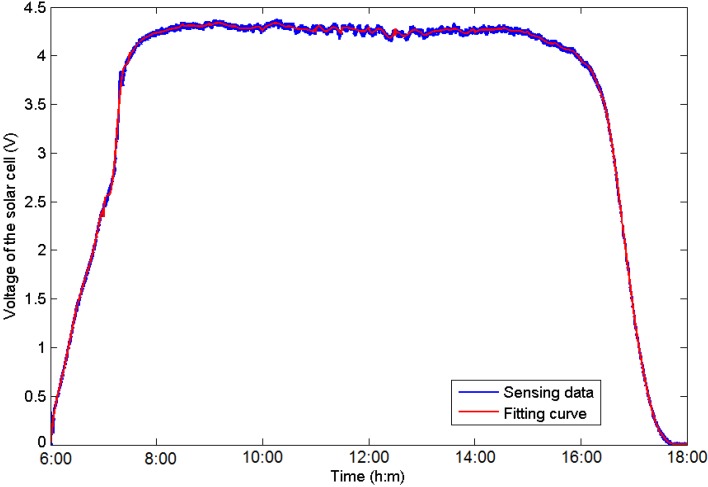
Example of the prediction procedure

Then, Equations (8)–(10) refresh the *a posteriori* state estimates of x at the update step based on the *a priori* estimate, which results in a new state estimate that lies between the predicted and the measured state and that has a better estimated uncertainty than either of them. The prediction and update steps are repeated at each round, and the new estimate and its covariance updating the prediction are used in the next iteration. This indicates that the filter requires only the last estimate value rather than the entire history to calculate a new state. The entire prediction procedure is shown in Figure 7. The A/D converter of the MCU on a WSN node measures the voltage of the solar cell in real time, and the EKF tool is then used to minimize the effect of noise on the sampling data. Then, the data are stored in an array for curve fitting, and the future voltage value is estimated using the curve parameters acquired at the last step. Considering the shadow conditions and the harvested energy in the future, the scheduling plan of the sensor node can be adjusted for a longer operation time. An example of this prediction procedure is presented in Figure 8. This evaluation is operated on the dataset from 6:00 to 18:00 on a sunny October day using the EKF tool and fifth-order polynomial function fitting. The operating window is selected as 2 hours for each unit, and the final MAD value is 18.9 mV. The evaluation reveals that the prediction procedure provides accurate estimations and is suitable for use at the sensor nodes.

### 4.3. Region Correlation and Shadow Detection

Because the WSN sensor nodes for environmental monitoring are widely used outdoors, they may be subjected to complex illumination patterns. Shadows produced by obstacles in the path of sunlight cover a small illuminative area on the field, which decreases the harvested energy compared with the surrounding areas. Furthermore, the shadow areas generated by trees or buildings move based on the angle of incidence of the sun throughout the day. When a sensor node initially enters the shadow area, its harvested energy suddenly decreases sharply. Thus, the coverage area of the shadow can be detected according to the differences of the harvested energy level between normal node and the node under the shadow. Furthermore, the movement and area change can be predicted by the movement history analysis.

Due to the absence of location equipment in popular WSN chips, the shadow area cannot be detected based on the exact positions of the nodes. However, the relative region correlation provides a method for approximately determining the area on an abstract level. The proposed algorithm clusters the nodes with low-level harvesting energies into a group of Density-based Spatial Clustering of Application with Noise (DBSCAN) [41,42] and then uses the bound of the group as the area of the shadow. DBSCAN is a density-based clustering method that creates clusters of objects in dense regions and divides the clusters into sparse regions. Unlike several other clustering methods, which can only determine clusters of convex shapes, DBSCAN can determine clusters of any shape, which is an advantage for detecting a variable shadow area with an unpredictable shape. Furthermore, another advantage of DBSCAN is its fast operating time, which is suitable for real-time applications.

**Figure 9:**
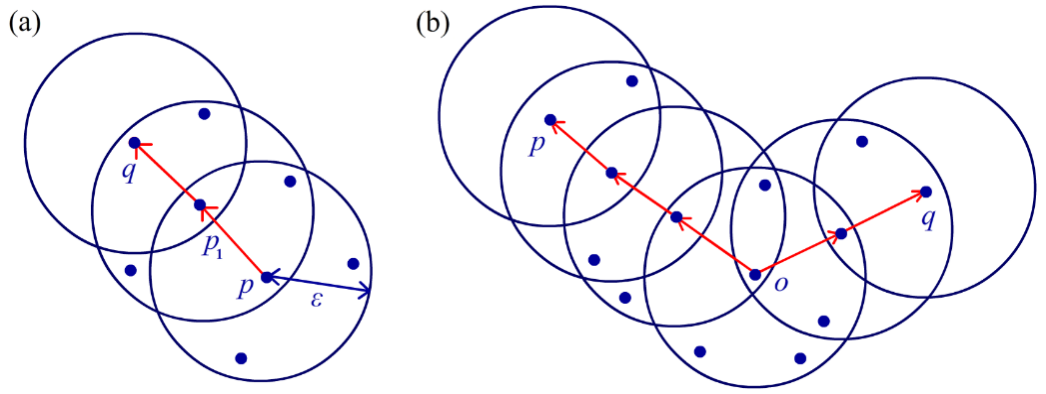
(**a**) *q* is density-reachable from *p*; (**b**) *p* and *q* are density-connected.

DBSCAN is briefly described in Algorithm 1. The density-connected relationship between two arbitrary objects is defined by two input parameters, ε and MinPts. A set including all objects within a distance ε from an arbitrary object p is defined as an ε-neighbour $N_{\varepsilon }(p)$. If the number of objects within $N_{\varepsilon }(p)$ achieves or exceeds MinPts, p is named a core object, and the object q in $N_{\varepsilon }(p)$ is defined as directly density-reachable from p. For two arbitrary objects p and q, if there exist objects p_1_,…,p_n-1_ such that p_i+1_ is directly density-reachable from p_i_ ($0\leq i<n,p_{0}=p,p_{n}=q$), then q is called density-reachable from p. For two arbitrary objects p and q, if there exists another object o such that both p and q are density-reachable from o, then p and q are called density-connected. Figure 9 illustrates an example of the relationship between p and q, where MinPts = 5. As indicated in Figure 9a, q is density-reachable from p, and p and q are mutually density-connected in Figure 9b.

**Algorithm 1.** DBSCAN procedure**Input**: a set of objects *S***Output**: a cluster of objects *C*Set all the objects in *S* to be unprocessed, C←Ø;**while** there is an unprocessed object in *S*  Choose an arbitrary unprocessed object p∈S and calculate $N_{\varepsilon }(p)$;  **if** $\left|N_{\varepsilon }(p)\right|\geq MinPts$   Build a new cluster *E* containing the only object *p* and add *E* into *C*;   $Child\leftarrow N_{\varepsilon }(p)-p$;   **while** there exist unprocessed or noise objects in *Child*    **for** each unprocessed or noise object q∈Child     Insert *q* into *E* and compute $N_{\varepsilon }(q)$;     **if**
$\left|N_{\varepsilon }(q)\right|\geq MinPts$      $Child\leftarrow Child\cup N_{\varepsilon }(q)-q$;     **end if**    **end for**   **end while**  **else**    Set the object *p* to be noise;  **end if****end while**

Algorithm 1 presents the DBSCAN procedure. Because it is necessary to compute the distance between each pair of objects, the time complexity of DBSCAN is O(n^2^), where n is the number of objects of the input set S. However, if there is a spatial structure for indexing all objects within a certain distance, the calculation time for ε-neighbour can be reduced to O(log n).

**Figure 10:**
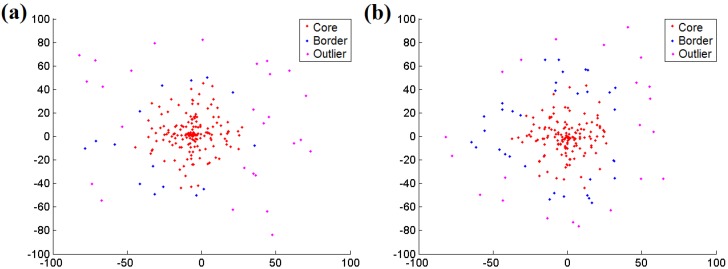
(**a**) An example of the DBSCAN algorithm addressing 200 random nodes; (**b**) Another example of the DBSCAN algorithm addressing 200 random nodes.

Therefore, the time complexity of the entire DBSCAN algorithm with a spatial index is O(nlog n), and it is suitable to be deployed on high-powered embedded equipment or workstations, e.g., the ARM Cortex series. Figure 10 illustrates two examples of the DBSCAN algorithm for addressing 200 random nodes in MATLAB. This proves that the DBSCAN clustering algorithm can efficiently identify the intensive area of suitable nodes and is competent for detecting the shadow region.

**Figure 11:**
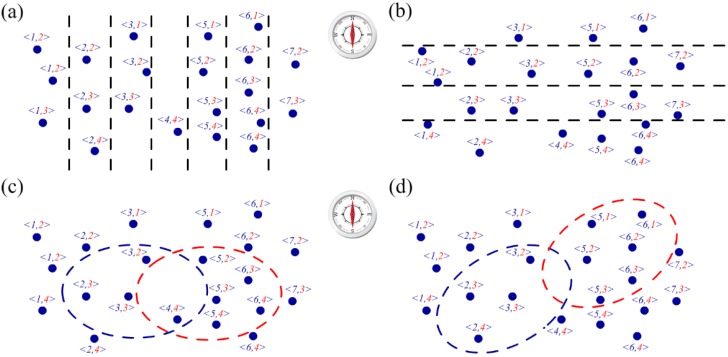
(**a**) Sensor node coded from west to east; (**b**) Sensor node coded from north to south; (**c**) Example of shadow moving from west to east; (**d**) Example of shadow moving by a certain angle.

The position and shape of the shadow region vary with the angle of incidence of the sun. To detect the moving position of the shadow, a two-tuple is defined to code the deployed node, as indicated in Figure 11. Figure 11a illustrates the sensor node coding from west to east using the first number in a two-tuple in blue. All of the sensor nodes in the field should be first divided into several groups using a set of virtual dividers based on their location coordinates recorded at deployment. The width of the gap between two virtual dividers is set by the user, and the nodes in the same gap can be coded from west to east sequentially. Figure 11b illustrates the coding style from north to south by the second number in the two-tuple in red, and the gap between two virtual lines is set by the designer. After coding, the movement of the shadow can be described by the change in the average codes. Let the average two-tuple <ƩFC/CN, ƩSC/CN> denote the current position of the shadow region, where FC represents the first number for each sensor node in the region coding from west to east, SC is the second number for each sensor node coding from north to south, and CN is the total number of nodes.

As indicated in Figure 11c, the shadow area moves from west to east and the average two-tuple changes from <3, 3> to <5.17, 3.33>, where the blue circle indicates the initial position and the red circle denotes the position after movement. Because the first code value difference 5.17 – 3 = 2.17, *i.e.*, greater than 1, the shadow is considered to move from west to east. Figure 11d illustrates another example of movement, where the average two-tuple changes from <2.5, 3> to <5.5, 2>. Because the differences in the first and second codes are both greater than 1, the shadow is considered to move from west to east and south to north simultaneously.

Moreover, the shape and area of the shadow region are described by the geographical coordinates recorded at the deployment of the nodes in the shadow group determined by DBSCAN. The shadow region can be viewed as an approximately circular area, and the radius is used to describe the size of the region. Thus, the predictions of the shadow movement and size variation are based on the value change of the two-tuple and the radius. The changing volume of the average number in the two-tuples reveals the moving direction, speed and future location of the shadow. The radius extracted for the coordinates of the nodes reveals the shape and size change of the entire shadow region.

### 4.4. Task Schedule

To schedule the task of the sensor nodes, the energy status of each node should first be estimated [14]. A structure with four elements, *i.e.*, <*E_p_(t), E_c_(t), P_t_, R_t_*>, can be used to describe the conditions at time *t*, where *E_p_(t)* and *E_c_(t)* represent the average predicted harvested energy defined in Equation (12) and consumed energy at time *t*, respectively; *P_t_* is the energy stored in the battery; *P_max_* is the maximum storage of the battery; and *R_t_* denotes the variation rate of the total energy, which can be calculated using Equation (11) as follows:

(11) $$R_{t}=[E_{p}(t)-E_{c}(t)]/P_{\max}$$

(12) $$E_{p}(t)=\sum_{t}^{t+win}E_{solar}(s)/win$$

where *E_solar_(s)* denotes the predicted harvested energy at time *s* acquired by curve fitting procedure in Section 4.1, *win* is a window width parameter for average computing which is often set to 30 *min* in our experiments. Thus, *E_p_(t)* is an average value for the predicted harvested energy of a short time interval.

Based on the harvesting and consumption conditions, the nodes can be separated into four different working statuses:

(1) *Redundant status (RS):* The RS occurs when the environment provides a considerable amount of energy to fully charge the battery and power the consuming device simultaneously. In other words, the battery cannot contain the large amount of energy harvested. In this mode, all of the operations on a sensor node, such as sampling, communication, and calculation, can be performed without limitations. When *E_p_(t) ≥ E_c_(t)* and *P_t_ = P_max_* on a node, it can be viewed as being in a RS.
(2) *Sufficient status (SS):* The SS mode is a pattern where the solar cell provides sufficient power to maintain the general operation of a sensor node and the residual energy can be used to charge the battery for future use. Under these conditions, energy accumulates in the battery over time, and the task scheduler has a relatively free choice. When *E_p_(t) ≥ E_c_(t)* and *R_t_ < P_max_*, a node is run under the SS*.*
(3) *Lack status (LS):* Under this pattern, in which *E_p_(t) < E_c_(t)*, the weak energy produced by the solar cell cannot single-handedly maintain the sensor node. To fill the energy gap, the battery has to supply a certain amount of energy. When operating under this pattern, the node is required to shutdown certain unnecessary functions to save energy.
(4) *Empty status (ES):* This mode is often activated at night or in bad weather, when there is no energy acquired from the solar cell and the battery fully powers the sensor node. Under this circumstance, most of the operation should be limited to maintaining the accessibility of the node as long as possible.

Based on the prediction of the environmental energy supply and the voltage condition of the battery, the transition between these four modes can occur automatically using the schedule program in Algorithm 2.

**Algorithm 2.** Transition procedure for four energy modes**Input:** Average predicted harvested energy: *E_p_(t)*, device consuming energy: *E_c_(t)*, residual energy level of the battery: *P_t_*, maximum battery capacity: *P_max_*, luminous intensity of the sunlight: *L_sun_*, luminous threshold: *L_threshold_***Output**: Working mode of the node**while** True   Delay *T_Delay_*;   Acquire *E_p_(t)*, *E_c_(t)* and *L_sun_*;   **if** *E_p_(t) ≥ E_c_(t)*    **if** *P_t_*
*< P_max_*     Mode: *Sufficient status (SS)*;    **else**     Mode: *Redundant status (RS)*;    **end if**   **else**    **if** *L_sun_≥ L_threshold_*     Mode: *Lack status (LS)*;    **else**     Mode: *Empty status (ES)*    **end if**   **end if****end while**

**Figure 12:**
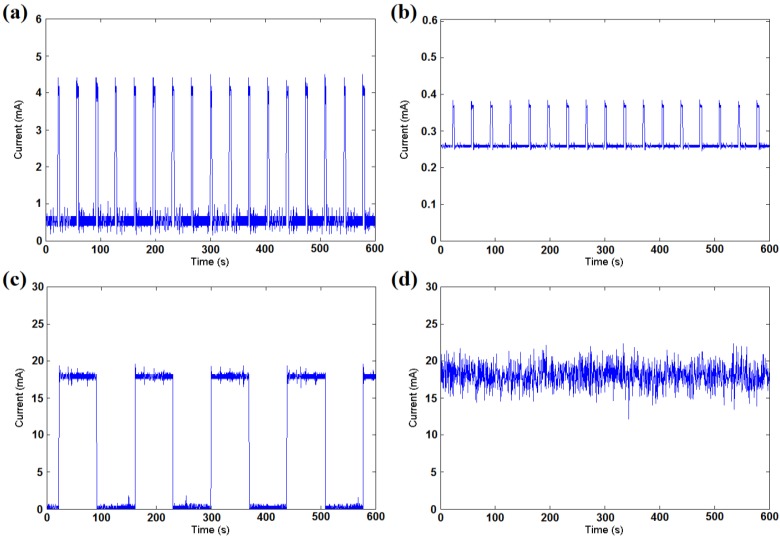
(**a**) Energy consumption of the 10% duty cycle with the LED on; (**b**) Energy consumption of the 10% duty cycle with light sampled once; (**c**) Energy consumption of the 50% duty cycle with idle listening; (**d**) Energy consumption of the 100% duty cycle with the sending of data.

After determining the working modes, the system can limit its energy use by adjusting the duty cycle. The duty cycle is a period parameter for sampling and transmission tasks. When a shorter duty cycle is set for the system, the data are sampled and transmitted back to the workstation more frequently. Furthermore, more energy is required in a certain time interval. Figure 12 illustrates the energy consumption of our hardware for several different components. Without loss of generality, three typical duty cycles of the sensor node are selected to test the energy pressure. It is determined that the energy consumed by different active components is also different, and the sending component uses approximately 1.5 mA more current than the idle listening component. The light sample component consumes nearly 0.4 mA, and the LED uses approximately 4 mA. Furthermore, the duty cycle plays an important role in the energy usage. The adjustment of the duty cycle based on the working mode determined by Algorithm 2 can reduce the energy use dynamically. The luminous threshold *L_threshold_* is a parameter for making a distinction between *LS* and *ES* mode. A high *L_threshold_* can prolong the working time of sensor node during a day and increase the energy consumption of battery. In order to maintain the sampling work at night, the parameter should be well set so that the harvested energy acquired in day time can be fully used. The setting principle depends on the real environment and hardware of the system. In our experiments, this parameter is set to 200 W/m^2^ or solar cell voltage of 0.5 V. The duty cycle can be set to fixed values for simple use, e.g., 100% in RS mode, 70% in SS mode, 30% in LS mode and 10% in ES mode. Additionally, it can be set to be a continuously adjustable value as a linear function of the prediction energy and the remaining battery level for precision.

## 5. Routing Optimization and Bottleneck Warning

The task schedule optimization intends to allow the node to be accessible as long as possible, but it is unable to extend the lifetime of the entire sensor network. Data transmission accounts for large amount of energy consumption in a wireless sensor network. Thus, the routing efficiency plays an important role in the energy control of the entire WSN. A suitable routing selection algorithm and a bottleneck warning mechanism are introduced to improve the energy balance of the network as a whole.

### 5.1. Sensor Node Cluster

Clustering is the first step for reducing the energy consumption for transmission. The sensor nodes within a certain physical distance can be organized as a group, and a clusterhead node is selected from them. The clusterhead gathers the sensing results from all of the other nodes in its cluster and then sends them to the next hop in the WSN. Due to the intensive data communication, clustering is a clearly proven method for saving energy. To distribute sensor nodes into each cluster, several algorithms have been proposed in previous studies [22,23,43,44]. These methods can be primarily divided into two categories: location methods, which are based on the geographical coordinates of the nodes before operating the network, and heuristic methods, which generate the cluster during network operation. Without GPS equipment at the sensor nodes, the network designer may need to arrange each node into a specific cluster by its placement on the map before performing it manually. Due to its low time complexity, this method is also implemented in our experiment.

**Figure 13:**
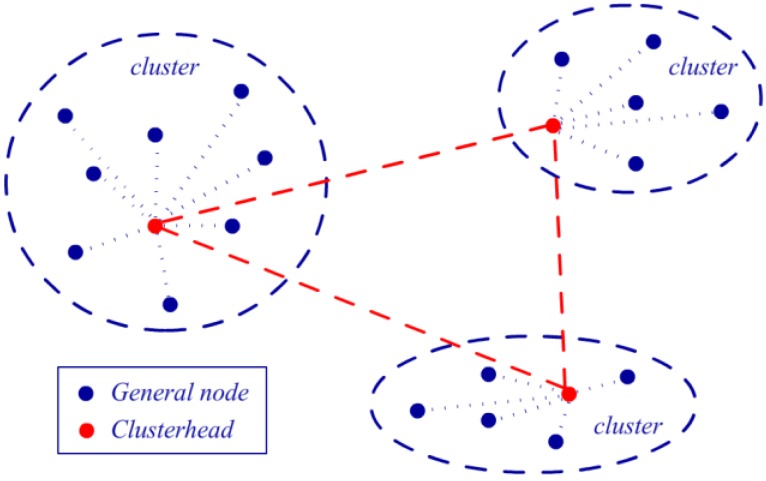
Illustration of a clustering mechanism.

Selecting the head node in a cluster after clustering is an important matter. A clusterhead involves the extra process of routing decision, data aggregation and communication. These tasks cost the head node more power compared with the general nodes. Thus, for nodes equipped with the same battery capacity, the clusterhead may lose its power significantly faster. Thus, the network should use a clusterhead rotation algorithm to address this problem by replacing the clusterhead after a period of time. An energy adaptive clusterhead selecting method is introduced to select the head node, and we use the average predicted harvested energy E_p_(t) at time t defined in Equation (12), device consuming energy E_c_(t) at time t and existing battery energy P_t_ at time t as the parameters for the decision. Equation (13) presents the formula for calculating the selection score of each sensor node as follows:

*V_score_ = P_t_ +γ·(E_p_(t) − E_c_(t))·Δt* (13)

where *γ* is a weighting coefficient, and Δt is the time interval for replacing the clusterhead. At the end of each time interval *Δt*, the system computes the voting score *V_score_* for each sensor node in a cluster and inserts it into a queue in descending order. Then, the first node in the queue with the largest voting score is selected as the clusterhead in this round, and it should gather information from all of the other nodes in this cluster and send them back together. To what extent the predicated harvested energy involved in the clusterhead selection depends on the parameter *γ*. A high value of *γ* is suitable for stable climate conditions, while a low one is adapted to changeable climate. Considering the weather condition at our experimental places, we chose *γ = 0.6* in our experiment according to preliminary testing. Figure 13 depicts an illustration of the clustering mechanism. All of the nodes in a WSN are divided into three clusters, and each cluster has its own clusterhead. The general nodes in a cluster are only allowed to communicate with their clusterhead, and the head node gathers sensor information from the general nodes and sends it to other clusters in the next hop.

### 5.2. Routing Optimization

After the clustering operation, the sensing information can be transferred as packets between the clusters until it reaches the gateway equipment. Generally, there is more than one technique for sending the packets, and the selection of the method is known as routing optimization. The construction of the WSN is not for mass data transmission but rather for sensing or monitoring. Thus, the primary target of the entire network is to execute its monitoring mission for as long as possible. The total transmission power is a commonly used metric considered in previous studies with the critical disadvantage of not directly reflecting the lifetime of the nodes in the WSN. Furthermore, the battery energy level of the nodes is a more accurate metric for describing the lifetime of the nodes. Thus, the Minimum Battery Cost Routing (MBCR) algorithm [45] was introduced for routing selection in previous studies using the battery level metric. Let $P_{t}^{i}$ denote the battery power level at time t for the node coded as i, and assume that a node’s willingness to forward packets is a function of its remaining battery energy level. Therefore, the less battery energy the node has, the more reluctant it is to transmit. The MBCR algorithm uses the reciprocal of the battery power as the cost $C_{t}^{i}$ to select the transmission path, which can be expressed using Equation (14) as follows:

(14) $$C_{t}^{i}=1/P_{t}^{i}$$

When the battery level decreases, the cost of the node will increase to obstruct route selection. The total cost of route j, consisting of N nodes, can be calculated using Equation (15). Therefore, to identify the transmission path with the maximum remaining battery level, we can choose a route s with a minimum battery cost using Equation (16), where E is the set containing all of the possible routes:

(15) $$T_{j}=\sum_{i=1}^{N}C_{t}^{i}$$

(16) $$T_{s}=\min\left\{T_{j}|j\in E\right\}$$

(17) $$T_{j}=\underset{i\in route\_j}{\max}C_{t}^{i}$$

Because the battery level is directly incorporated into the routing method, this metric prevents the sensor nodes from being overused. Moreover, if all of the nodes have similar battery energy levels, the method will select a shorter-hop route. However, because only the summation of battery use is considered, the network route may pass a node with a battery energy shortage on a rich total energy path. Furthermore, the battery of this node will be eventually exhausted, and the node will become inaccessible because of route interruption. To make sure that no node will be overused, Equation (15) can be transformed into Equation (17) to select the least value for battery energy on the path as the cost of the entire route. This transformation produces a new method known as Min-Max Battery Cost Routing (MMBCR) [46]. This new metric always attempts to avoid the route with nodes that have the least battery energy, and it can be used more fairly than the previous method. However, further study revealed that this new metric can consume more power in transmitting user data from a source to a destination because there is no guarantee of a minimum total transmission power, thus reducing the lifetime of all of the nodes. In summary, selecting between MBCR and MMBCR should involve considering both the application environment and the actual energy consumed.

Furthermore, the two methods mentioned above only use the remaining battery energy as the metric; however, the harvested energy dynamically influences the battery level. Thus, the prediction of the harvesting conditions should also be considered. Additionally, Equation (14) can be improved as Equation (18), where λ is a weighting coefficient, similar to γ in Equation (13), and where ΔT is the time interval for updating the route. Hence, the corresponding proposed methods are known as Minimum Battery Cost Routing with Harvesting Prediction (MBCRHP) and Min-Max Battery Cost Routing with Harvesting Prediction (MMBCRHP):

(18) $$C_{t}^{i}=1/[P_{t}+\lambda \cdot (E_{p}(t)-E_{c}(t))\cdot \Delta T]$$

**Figure 14:**
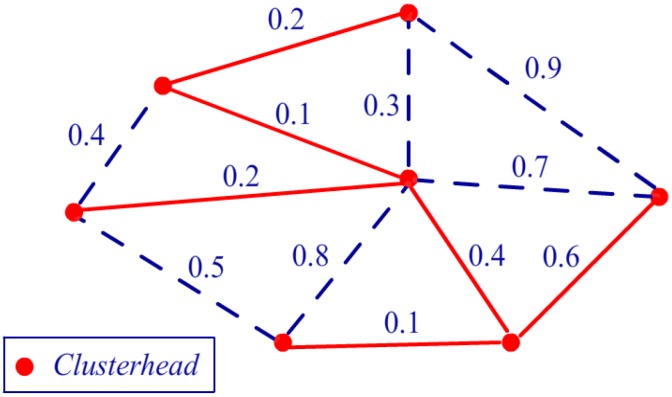
Illustration of the route selection algorithm.

After selecting the metric, the system needs to determine the path on the map. To solve this problem, a minimum spanning tree algorithm can be introduced. A minimum spanning tree of an undirected graph G is a tree formed from graph edges that connects all of the vertices of G at the lowest total cost. Furthermore, this tree can be used as the route of data transmission in the network. The Prim [47,48] algorithm is a classical method used to build the minimum spanning tree. At any point in the Prim algorithm, there is a set of vertices that have already been included in the tree; the rest of the vertices have not. The algorithm then determines, at each step, a new vertex to add to the tree by selecting an edge (u, v) such that the cost of (u, v) is the smallest among all edges where u is in the tree and v is not. Thus, each step adds one edge and one vertex to the tree. Then, after all of the vertices are added to the tree, the algorithm finishes its task, and the transmission route is built. Figure 14 presents an illustration of the route selection algorithm where the Prim algorithm is run on clusterheads in the WSN.

### 5.3. Bayesian Networks and Bottleneck Warning

After node clustering and route selection, the sensing data can be gathered from each node and transmitted to the workstation. However, similar to a crowded road in the city, there are certain clusterheads in the network that require more transmission work compared with others, such as the centre node in Figure 14. This type of node is often called a bottleneck node, and it is typically located at the connection of two or three clusters. Even if this type of node initially has more power than the general nodes, its transmission consumption will be several times greater than that of the others. Finally, the bottleneck node will have no energy left, and the connection will be interrupted. If the bottleneck nodes can be detected in an evaluation before the system operates, the mechanism of double clusterheads in the bottleneck cluster can be used to improve the situation. It should be indicated that the traditional clustering algorithms [25,26] also have the latent mechanisms to rotate the clusterheads to avoid overuse, but these mechanisms mainly involve the factors inside a cluster. However, the bottleneck problem defined in our work is presented as a transmission problem caused by structure of the network, and it is the problem among different clusters. Actually, there may be a cluster on the only way by which other clusters transfer their data to the workstation. And then the clusterhead in this key cluster will take more transmission work than clusterheads in other ones. Thus, our bottleneck warning algorithm is mainly proposed to improve this problem outside a certain cluster. Bayesian networks (BNs) [49,50] are probabilistic graphical models used for classification and are useful for predicting complex engineering problems, such as traffic congestion [51]. Because of their powerful reasoning ability, they are introduced to evaluate the bottlenecks in our study.

A BN formally consists of Directed Acyclic Graphs (DAGs). It is an easy method for representing the structure of a probabilistic model and the conditional independence relationships between the nodes. The nodes in a BN are used to represent random variables, and the arrow linking two nodes, which is quantified by their conditional probabilities, indicates the dependency between them. For example, an arrow from node A to B indicates the conditional dependence of B given A, which can be quantified as P(B|A). Thus, once the structure of the model is specified and the conditional probability is set for each link, the BN can be fully described by a set of parameters **μ**, which indicate the conditional probabilities between the nodes. For each node, a Conditional Probability Table (CPT) is available that records its relationship with its parent nodes. The BN use Bayesian methodology to quantify the changes in the node CPT values for introducing new evidence or updating old evidence. This procedure is known as “uncertainty propagation” or “belief updating”, and an existing algorithm allows its efficient computation [49].

Before the prediction procedure of the BN, the network should learn to estimate the sets of conditional probabilities. The learning procedure aims to compute the conditional probabilities between the nodes using the training data, *i.e.*, it aims to construct the CPTs for each node in the network. This procedure can be accomplished using the Expectation Maximization (EM) algorithm [52], an iterative algorithm that provides the new maximum likelihood estimate (MLE) of model parameters **μ** when certain variables are missing at random. The EM algorithm is described in Algorithm 3 [49].

**Algorithm 3.** Expectation maximization algorithm for BN learningStep 0:Initiate ***μ*** to ***μ^0^*** and set ***μ^t^*** = ***μ^0^*** and then continue;Step 1:To compute ***μ^t+1^*** once ***μ^t^*** is known, complete Step 2 and Step 3;Step 2:Expectation step: compute the data set based on ***μ^t^***:a)Compute the conditional probability distribution of missing values v* using the Bayesian formula as follows:  $P(v*|v,\mu^{t})=\frac{P(v*,\mu^{t})P(v|v*,\mu^{t})}{\sum_{v*}P(v*,\mu^{t})P(v|v*,\mu^{t})}$, where ***v*** is the set of observed values.b)Obtain the fractional value by assigning a weight, given as $P(v*|v,\mu^{t})$, to the missing values v*, and add the value into the incomplete data set to construct the completed data set.Step 3:Maximization Step: To obtain the MLE of the model parameters ***μ^t+1^***, compute the set of parameters that maximize the likelihood of the completed data set acquired in Step 2b.Step 4:If convergence is obtained, the algorithm stops; if not, make *t* = *t+1* and ***μ^t^* = *μ^t+1^***, and return to Step 1.

After the learning step, the Bayesian network can be used to predict the unknown data. Hence, the prediction phase in the BN is typically referred to as probabilistic inference [53]. The inference yields the posterior probability distributions based on the given evidence, and the posterior probability reveals the problem results that need to be solved. There are two typical inferences used in BNs, exact and approximate. The Junction Tree (JT) algorithm [54] is one of the most popular algorithms used for exact inference in BNs, and it is based on a thorough analysis of the connection between graph theory and probability theory. The JT algorithm can be summarized in three primary steps in Algorithm 4 [49]. In this study, we used BNs and the JT algorithm to predict the bottleneck clusterhead in the sensor network on a workstation before each time interval. Then, the double clusterhead mechanism can be used to avoid energy overuse at the bottleneck node.

**Algorithm 4.** Junction tree algorithm for BN inferenceStep 1: Construct a junction tree based on the existing BN;Step 2: Propagate messages along the junction tree using a message passing algorithm;Step 3: Answer queries when evidence is introduced.

**Figure 15:**
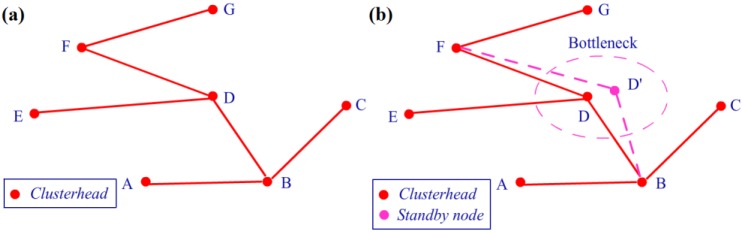
(**a**) Illustration of the routing situation. (**b**) Illustration of double clusterheads.

In the first step of this application, the CPT should be built for each clusterhead using the learning procedure in the network. For example, consider the routing situation presented in Figure 15a, which is generated from Figure 14 using the Prim algorithm. There are seven clusterheads from A to G. We use three nodes, A, B, and D, to illustrate how to build a CPT. Table 3 presents an illustration of the historical statistics of the clusterhead data flow, and the value 5 appears five times in the first row for node A at t_2_, t_3_, t_5_, t_7_, and t_8_, while value 3 appears three times at t_1_, t_6_, and t_10_. All of the values are counted in Table 4, and the CPT can be calculated for node B in Table 5 using Algorithm 3. After the CPT building procedure is performed for each node in the network, Algorithm 4 is used for BN inference.

**Table 3:** Illustration of the previous statistics of the clusterhead data flow.

Data flow	Time	t_1_	t_2_	t_3_	t_4_	t_5_	t_6_	t_7_	t_8_	t_9_	t_10_

Node											
A		3	5	5	2	5	3	5	5	6	3
B		5	4	6	6	3	6	3	6	4	5
D		3	6	5	8	7	5	8	5	7	6

**Table 4:** Illustration of the statistics of the data flow for node A.

Data Flow in Unit Intervals	Times of the Data Flow
2	1
3	3
5	5
6	1

**Table 5:** Illustration of the conditional probability table for node B.

	A	2	3	5	6
P(B|A)					
B					
**3**		1	0.50	0	0
**4**		0	0.50	0.25	0
**5**		0	0	0	1
**6**		0	0	0.75	0

At the inference stage, new evidence is transferred to the father and sons of the node and then gradually broadcasted along the route of the network. An update is performed at each node in the CPT, and the posterior probability of each possible transmission flow can be acquired. Based on the probability of a large flow, we can detect the risk of congestion in data transmission by comparing the value to a preset threshold. In our system, the threshold is set to three times as large as the average transmission load of all clusterheads. Before the system is formally used, a testing procedure is run on the whole network to evaluate the transmission load for each clusterhead. Then, the relevant clusterhead with a transmission load above the threshold can be viewed as the bottleneck node in the sensor network, and the mechanism of double clusterheads can be used to improve the situation. Figure 15b presents an illustration of the double clusterhead mechanism. The clusterhead node D is detected as the bottleneck node in the network, and D’ is selected as a standby node in the same cluster using a queue to share the data transmission task of D. The queue is built according to the *V_score_* defined in Equation (13), which is sorted in descending order. The second element of the queue is selected as the standby node. This mechanism will reduce the energy load of node D and prolong the lifetime of the entire network. The relevant experiments are implemented on real-time hardware, and the results are presented in the next section.

## 6. Implementation and Experiments

**Figure 16:**
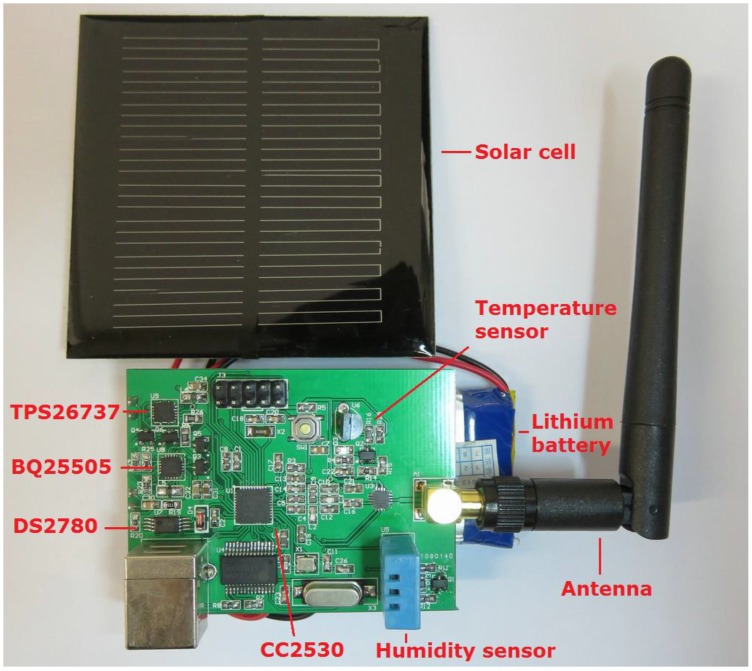
Hardware illustration of the sensor node.

The sensor network is implemented on the TI CC2530 platform, which is a true system-on-chip (SoC) solution for the IEEE 802.15.4, Zigbee and RF4CE applications. The CC2530 combines a leading RF transceiver with an industry-standard enhanced 8051 MCU. A TI BQ25505 chip with TPS62737 is used to manage the harvested energy on-board. This is an integrated Nano-Power management solution that is well suited for meeting the special needs of ultra-low power applications. It is specifically designed to efficiently acquire and manage the microwatts (μW) to milliwatts (mW) of power generated from solar or thermal electric generators. Moreover, a MAXIX DS2780 chip is used to measure the remaining energy level of the battery. The DS2780 is a 16-bit professional measure IC for estimating the available capacity for rechargeable lithium batteries. The hardware illustration of the sensor node is presented in Figure 16.

**Figure 17:**
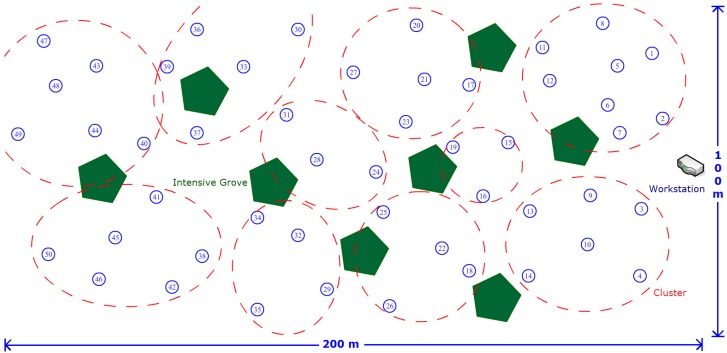
Illustration of the deployment of TSEP sensor nodes.

**Figure 18:**
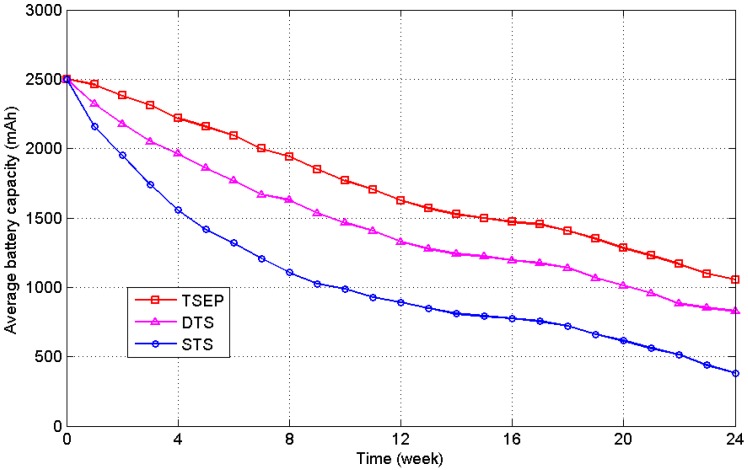
Average battery energy levels of the three experimental groups.

All of the experiments were performed in Fuzhou, a city in Southeast China, from April to November to ensure ample sunshine. The harvesting prediction experiments were conducted in three groups, and each group included 50 sensor nodes to construct a WSN. Each sensor node was programmed under a C51 compiling system at the protocol level based on the clustering and routing algorithm presented in this study. The three groups were deployed in similar places and used different energy management methods for task scheduling: (1) task scheduling with energy prediction (TSEP): task scheduling based on the harvested energy prediction using the EKF filter proposed in Section 4; (2) dynamic task scheduling (DTS): task scheduling that depends only on the remaining energy level of the battery; and (3) static task scheduling (STS): sampling and sending during a fixed time interval of 10 minutes without energy feedback, where the night duty cycle is extended to 30 minutes. In order to employ task scheduling in TSEP and DTS groups, the *L_threshold_* defined in Algorithm 2 is set to 200 W/m^2^ or solar cell voltage of 0.5 V. The voltage of the solar cell and residual battery energy were sampled at a 5-minute interval, and the consuming of energy was estimated by the differences between two neighbour sampling values. Piecewise fitting with the extended Kalman filter of 60-min section was employed in the TSEP group to predict the harvested energy. The duty cycles for dynamic task scheduling for each sensor node defined in Algorithm 2 are set as below: 5 min for *SS* mode, 10 min for *RS* mode, 20 min for *LS* mode and 30 min for *ES* mode. When there is not sufficient energy harvested from sunlight, the sensor node will be slowed down to decrease energy consumption. Furthermore, The clustering and routing optimization were run on all three groups with *γ = 0.6*, *Δt = 1 h* in Equation (13) and *λ = 0.6*, *ΔT = 3 h* in Equation (18), which means the clusterhead rotation time interval is 1 h and the routing valid period is 3 h. The bottleneck warning mechanism was used to improve the transmission structure of the network. The threshold is set to 3 times as large as the average transmission load of all clusterheads. Figure 17 shows the deployment of TSEP sensor nodes in a 200 m × 100 m garden area. The green pentagon represents the approximate location of intensive grove in this area. There are also some small bushes in the region and it is hard to point them out one by one. The 50 sensor nodes are scattered in the region avoiding intensive grove manually, and numbered from east to west. The red circles indicate a probable situation for clustering acquired by our method in experiment, and the place of workstation is also shown. The deployment of DTS and STS groups is according to the TSEP, and the area is also 200 m × 100 m. However, the location of intensive groves is similar but not the same as it is difficult for us to find two garden areas with the same tree locations. The experiments were performed for 24 weeks from April to September and results are provided in Figure 18.

At the end of the 24-week test period, TSEP, DTS, and STS have an average remaining energy of 1057 mAh (42.28% of 2500 mAh), 823 mAh (32.92%) and 381 mAh (15.24%), respectively. It is determined that TSEP uses 1443 mAh of energy during the entire procedure, which is 68.1% of the 2119 mAh of energy used by STS. It is confirmed that the nearly 30% of the energy consumption was saved by the prediction and dynamic task scheduling mechanisms, and the EKF filter improved the precision of this procedure. The extra prediction mechanism caused the TSEP to use 9.4% less energy compared to DTS without the mechanism. Furthermore, Figure 18 shows that the energy difference between TSEP and the STS rapidly increased in the first 8 weeks because of the rainy weather conditions in spring in southern China. Additionally, July and August have the largest solar energy radiation in the year, and the difference decreased during this time. It has been proven that the dynamic scheduling mechanism with energy prediction plays an important role in the energy management of the WSN, especially under complicated weather conditions. The differences are probably caused by the task scheduling mechanism employed in this study, as it can slow duty cycle of nodes in bad weather or under shadow environment promptly.

**Figure 19:**
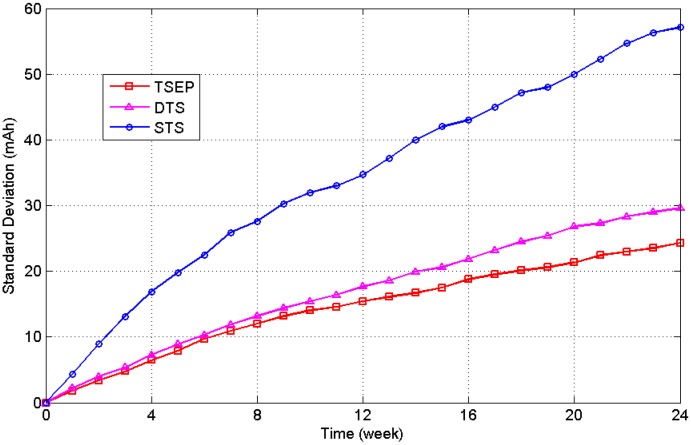
Standard deviation of the battery levels of the experimental groups.

Figure 19 depicts the standard deviation of the battery level for the three experimental groups, which indicates the balancing ability of the algorithm for all of the sensor nodes. Harvested solar energies at different locations are different, and without the task scheduling mechanism the energy differences between nodes will be accumulated over time.

After the 24-week test, STS, DTS, and TSEP had standard deviations of 57.1, 29.6 and 24.3 mAh, respectively. The standard deviation of TSEP was only 42.6% of that of STS. It is proven that the energy prediction and dynamic task scheduling mechanisms can balance the energy consumption in the WSN. The dynamic task scheduling can reduce the loss rate of sensor nodes in the whole network, as a results, the robustness of the system can be increased and the sensoring area can be kept during the work time.

**Figure 20:**
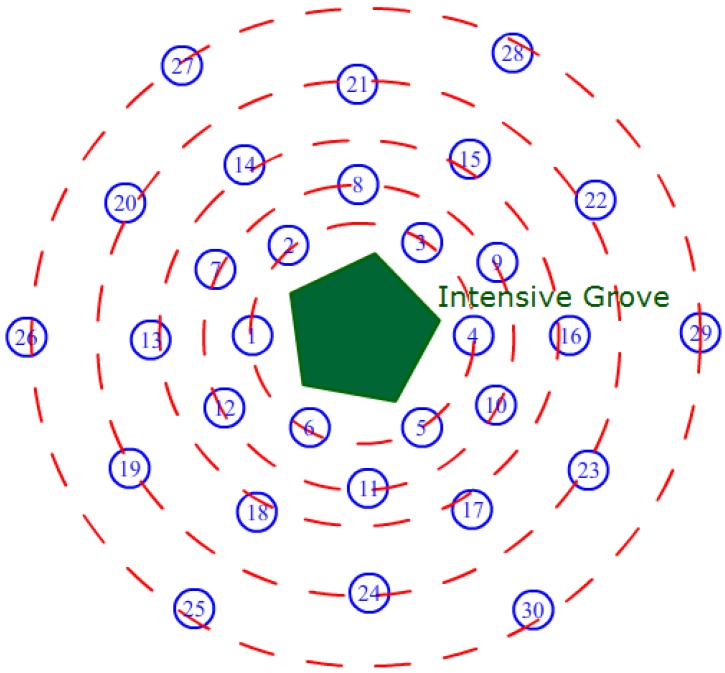
Illustration of deployment for the shadow experimental groups.

To test the effects of the shadow detection mechanism, we used 60 nodes in two groups to explore the energy management for a period of 60 days. Each group included 30 sensor nodes deployed in the region of a grove shadow as Figure 20 shows. In this experiment, we changed the node number to 30 in order to have the circled deployment, and we think it’s enough. It can be found in Figure 20 that the sensor nodes are uniformly deployed on circles around the intensive grove, and the radii of the circles are 1, 2, 3, 5, and 8 m respectively from the inside to the outside.

All sensor nodes are distributed by an angle of 60 degrees on the circles, which can relate to the shadow area in all directions. In fact, the size and shape of the shadow changed during the day based on the angle of incidence of the sun, and there were different nodes in the exact region of the shadow at different times. Hence, it is difficult to estimate the energy situation of an exact sensor node using the shadow detecting method. The average battery energy level can be viewed as an approximate measure for shadow detection because the duty cycle is reduced by the algorithm to save energy based on the shadow detection results. Another measure is the detection rate *DRate*, as defined in Equation (19), where *Num_shadow_* represents the total number of nodes in the shadow region and *Num_right_* is the number of nodes detected correctly.

(19) $$DRate=Num_{right}/Num_{shadow}$$

**Figure 21:**
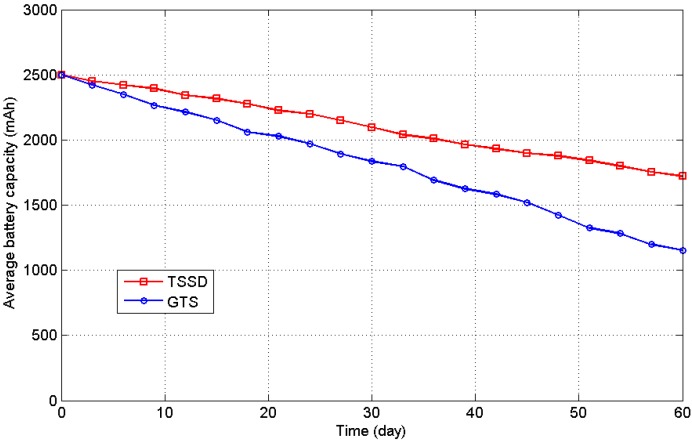
Average battery energy levels of the shadow experimental groups.

During the experimental period, the energy data were gathered through the network every 6 days, and the detection rate was calculated using the detecting algorithm and the shadow region observation. When a node’s harvested energy is 50% less than the average value of the entire network, it can be assumed to be in the shadow region. In the test, the Task Scheduling with Shadow Detection (TSSD) group used the task scheduling, including harvested energy prediction and shadow detection proposed in this study, whereas the Group with Task Scheduling (GTS) only used the general task scheduling, considering the remaining battery level. Figure 21 presents the experimental results; after the 60-day experiment, the GTS had an average battery capacity of 1154 mAh, whereas the TSSD group had an average battery capacity of 1721 mAh and used 42.1% less energy than the GTS. The detection rate was calculated by observation three times daily, and the average value was obtained after the test. The final *DRate* value was 82.7%, which indicates that most of the nodes in the shadow region were detected correctly using the proposed method.

For the clustering and routing algorithms, we have introduced new metrics for clusterhead selection and a bottleneck warning mechanism to avoid overuse of the clusterheads. To explore the effects of these methods, we conducted an experiment with two groups. A measure of the loss rate defined in Equation (20) was used to follow the effects of the algorithm, where *Num_clusterhead_* represents the total number of clusterheads in the WSN and *Num_lost_* is the number of lost nodes:

(20) $$LRate=Num_{lost}/Num_{clusterhead}$$

**Figure 22:**
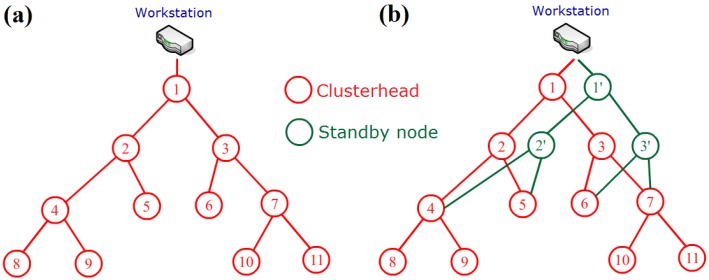
(**a**) Illustration of transmission topology of SCM group; (**b**) Illustration of transmission topology of DCM group.

When a node’s battery level is 10% below its total capacity, it is considered to be lost. Two groups of 50 nodes each, which used a single clusterhead method (SCM) and a double clusterhead method (DCM) with bottleneck warning, were used to test for differences. The sensor nodes of each group are exposed to sunlight in a 200 m × 100 m open space without trees for acquiring harvested energy fully. Figure 22 shows the transmission topology of SCM and DCM groups respectively, and clusterheads are arranged intentionally to construct a transmission link with congestion risk. For simplicity, the other general nodes are omitted to be shown. Furthermore, at the beginning of experiment, the SCM group uses fixed clusterheads which can not be replaced until their energy is exhausted. When its residual energy is lower than 10%, the clusterhead is marked as the lost node and replaced by other general node in its cluster. However, the DCM group employs the bottleneck warning mechanism proposed in this paper which arranges standby clusterhead for node 1, 2 and 3 notated as 1’, 2’ and 3’ in Figure 22b. Both two groups use dynamic task scheduling methods proposed in this paper but do not employ the clusterhead rotation method. The duty cycles for each sensor node are set as below: 10 min for *SS* mode, 20 min for *RS* mode, 40 min for *LS* mode and 60 min for *ES* mode. Extension of the duty cycle is in order to observe the phenomenon more clearly. Other necessary parameters are the same as the experiment of task scheduling motioned above. The experiments are performed for 8 months from April to November to consume the energy of clusterheads fully, and then the differences between mechanisms can be shown obviously.

**Figure 23:**
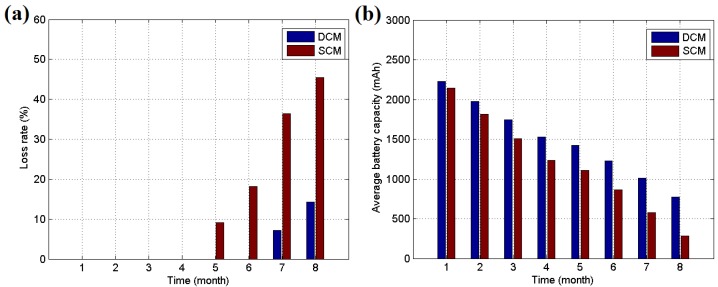
(**a**) Loss rate of the clusterheads during the experiment; (**b**) Average battery capacity of the clusterheads during the experiment.

Figure 23 presents the results of the experiment over 8 months. It is determined that after this time months, the SCM loss rate reached 45.5%, whereas the DCM loss rate was only 14.3%. Additionally, the DCM has an average battery capacity of 773 mAh, which is greater than the corresponding value of 283 mAh for SCM. It is evident that without the clusterhead rotation method the bottleneck warning mechanism can share part of the workload for the clusterhead to extend its lifetime, and it may prolong the total lifetime of the entire network. The WSN using fixed clusterheads are often deployed for simple applications due to its easy implementation. So the bottleneck warning method and double clusterhead mechanism can be employed in these situations for improving the robustness of WSN. However, for advanced usage, the clusterhead rotation mechanism can achieve better performance in sophisticated environment. A 90-day short-term test is performed to explore the performance of the double clusterhead mechanism under clusterhead rotation setting. The circumstance in this test is the same as before, except the clusterhead rotation mechanism is added into SCM and DCM groups. Hence, the single clusterhead method with rotation (SCMR) and the double clusterhead method with rotation (DCMR) are built for testing.

After 90-day testing, the average battery capacity for these two groups are shown in Table 6. It is found that during the whole 90-day test the DCMR costs 770 mAh average battery capacity which is 97.7% of the one cost by SCMR. The performance of double clusterhead method with bottleneck warning is not obviously better than the traditional single clusterhead method for short-term applications. But its advantages can be accumulated as time goes on. During that 90-day test, the energy cost by the double clusterhead mechanism is 2.3% lower than the traditional method. So in a longer application, more energy can be saved. According to our analysis, the saving energy is probably caused by two reasons: (1) the double clusterhead mechanism has higher efficiency of energy use; (2) the standby clusterhead shares transmission data from clusterhead on Medium Access Control (MAC) layer to avoid frequent collision. In Equation (2), we have introduced *θ* as a parameter to describe the charging efficiency. When we convert harvested electric energy to chemical energy in battery, part of the energy will be lost according to *θ*. So if the nodes are able to use the harvested electric energy as possible as they can and avoid storing them into battery, the efficiency of energy use can be improved. Let *E* represent the harvested energy from solar cell on a clusterhead, and *e* denote the transmission energy consumption for it. So the formulas for three energy circumstances in the above test are shown in Table 7 according to Equation (2). Due to the fact that there may be two clusterheads in a same cluster of DCMR, we add a general node into calculation of SCMR for fairness. When *E ≥ e*, the harvested energy is sufficient for transmission work on the clusterhead, and the redundant energy should be stored in battery.

**Table 6:** Results of the clusterhead selection test with rotation mechanism.

Time (days)	Average Battery Capacity for DCMR (mAh)	Average Battery Capacity for SCMR (mAh)
0	2500	2500
10	2418	2418
20	2329	2328
30	2235	2233
40	2148	2145
50	2067	2062
60	1991	1983
70	1909	1898
80	1823	1808
90	1730	1712

**Table 7:** Formulas for three energy circumstances in test.

Method	Node Situation	Harvested Energy	Energy Request	*Saved Energy* *When E < e/2*	*Saved Energy* *when e/2 ≤ E < e*	*Saved Energy* *When E ≥ e*
SCMR	1 clusterhead, 1 general node	*E*, *E*	*e*, *0*	*(E − e)*, *θE*	*(E − e)*, *θE*	*θ(E − e)*, *θE*
DCMR	1 clusterhead, 1 clusterhead	*E*, *E*	*e/2*, *e/2*	*E − e/2*, *E − e/2*	*θ(E − e/2)*, *θ(E − e/2)*	*θ(E − e/2)*, *θ(E − e/2)*

(21) *A_1_ =(E − e) + θE ;B_1_ =2(E − e/2)*

(22) *A_1_-B_1_ = (E − e) +θE − 2(E − e/2) = (θ− 1)E<0*

(23) *A_2_ =(E − e) + θE ;B_2_ =2θ(E − e/2)*

(24) *A_2_−B_2_ = (E − e) +θE − 2θ(E − e/2) = E – e +θE − 2θE +θe = (1 −θ)E − (1 −θ)e = (1 −θ)(E − e) < 0*

(25) *A_3_ =θ(E − e) +θE ; B_3_ = 2θ(E − e/2)*

(26) *A_3_ − B_3_ =θ(E − e) +θE − 2θ(E − e/2) = 0*

As Table 4 shown, when *E < e/2*, the saved energy for the two nodes in SCMR (1 clusterhead and 1 general node) can be calculated by *A_1_* in Equation (21), and the saved energy for the two clusterheads in DCMR can be calculated by *B_1_*. Due to *θ < 1*, the A_1_-B_1_ is below zero as Equation (22), and then A_1_ < B_1_. When *e/2 ≤ E < e*, the formulas for calculating saved energy in SCMR and DCMR are shown in Equations (23) and (24). For *θ < 1* and *E < e*, the A_2_-B_2_ is also below zero according to Equation (24), and then A_2_ < B_2_. However, when *E ≥ e*, the calculation for saved energy is shown in Equations (25) and (26), and A_3_ = B_3_. Based on the above, it has been known that when *E < e* the DCMR can acquire more saved energy in battery than SCMR, and when *E ≥ e* the saved energy for these two methods are equal in formula. Thus, the double clusterheads can improve the efficiency of energy use for harvested energy under certain circumstance. However, employing double clusterheads in a cluster results in an extra computational load for the system, so the standby clusterheads should be kept in an appropriate number. To avoid frequent collision on MAC layer is another reason for saving energy. The frequent collisions produced by large data transmission will make the general nodes to access clusterhead for more than once in a duty cycle, and that will lead to the increase of energy consumption.

## 7. Conclusions

In theory, an energy-harvesting system should allow sensor nodes to have a potentially infinite lifetime. However, the harvested energy is intermittent in practice, and it may not even be sufficient to constantly satisfy the demand of outdoor applications. Thus, the energy harvesting procedure should be modelled, and the task schedule of the sensor node should be optimized to prolong the lifetime of the WSN as much as possible. To obtain a higher energy efficiency, a harvesting prediction algorithm with shadow detection is proposed in this report. Furthermore, advanced clustering and routing methods are introduced to save energy for communication. A real-time system is implemented on a TI CC2530 platform, and the experimental results demonstrate the improvement in these algorithms.
